# Long-standing LPG subsidies, cooking fuel stacking, and personal exposure to air pollution in rural and peri-urban Ecuador

**DOI:** 10.1038/s41370-020-0231-5

**Published:** 2020-05-15

**Authors:** Carlos F. Gould, Samuel B. Schlesinger, Emilio Molina, M. Lorena Bejarano, Alfredo Valarezo, Darby W. Jack

**Affiliations:** 1 Department of Environmental Health Science, Columbia University Mailman School of Public Health, New York, NY USA; 2 Independent Consultant, Quito, Ecuador; 3 Department of Mechanical Engineering, Universidad San Francisco de Quito, Quito, Ecuador

**Keywords:** Clean cooking, Fine particulate matter, Air pollution, Personal exposure

## Abstract

Ecuador presents a unique case study for evaluating personal air pollution exposure in a middle-income country where a clean cooking fuel has been available at low cost for several decades. We measured personal PM_2.5_ exposure, stove use, and participant location during a 48-h monitoring period for 157 rural and peri-urban households in coastal and Andean Ecuador. While nearly all households owned a liquefied petroleum gas (LPG) stove and used it as their primary cooking fuel, one-quarter of households utilized firewood as a secondary fuel and 10% used induction stoves secondary to LPG. Stove use monitoring demonstrated clear within- and across-meal fuel stacking patterns. Firewood-owning participants had higher distributions of 48-h and 10-min PM_2.5_ exposure as compared with primary LPG and induction stove users, and this effect became more pronounced with firewood use during monitoring.Accounting for within-subject clustering, contemporaneous firewood stove use was associated with 101 μg/m^3^ higher 10-min PM_2.5_ exposure (95% CI: 94–108 μg/m^3^). LPG and induction cooking events were largely not associated with contemporaneous PM_2.5_ exposure. Our results suggest that firewood use is associated with average and short-term personal air pollution exposure above the WHO interim-I guideline, even when LPG is the primary cooking fuel.

## Introduction

Exposure to household air pollution (HAP) from the inefficient combustion of biomass fuels is a leading health risk, contributing to an estimated 1.8 million premature deaths each year [1]. Large-scale transitions to cleaner cooking promise substantial health [2, 3], environmental [4], climate [5, 6], and gender empowerment benefits [7–9]. While recent efforts to mitigate this burden of disease have focused on promoting clean cooking fuels, these remain prohibitively expensive or inaccessible in many rural parts of low- and middle-income countries where the impacts of traditional biomass cooking are greatest [10, 11]. As a result, examples of widespread long-term clean cooking fuel use in disadvantaged populations of low- or middle-income countries are rare, and associated HAP reduction and attendant health benefits are poorly characterized.

Ecuador is a unique case study where two overlapping nationwide clean cooking fuel subsidy programs have made clean cooking options accessible to most of the population [12]. Now, more than 90% of Ecuadorian households cook with clean fuels—primarily liquefied petroleum gas (LPG)—instead of firewood and kerosene, which were the dominant household cooking fuels through the 1980s.

In previous work surveying more than 1000 households in rural and peri-urban Andean and coastal Ecuador, we have shown that LPG is nearly universal as the primary household cooking fuel [12, 13]. Still, approximately half of rural households and two-fifths of peri-urban respondents continue to use firewood. We have also previously found limited induction stove ownership and use, though up to 20% of households owned an induction stove in some study communities. The implication of these persistent cooking fuel stacking patterns for personal air pollution exposure is unknown.

The primary goal of the present study is to estimate the personal PM_2.5_ exposure of primary cooks in rural and peri-urban households in coastal and Andean Ecuador, as well as their use of different cooking fuels. We deploy well-validated instruments to estimate average 48-h and 10-min personal PM_2.5_ exposure and measure all household use of cooking fuels during a 48-h monitoring period in 117 rural and 40 peri-urban households. We also continuously monitored traditional firewood stove use for several months in eight households to assess long-term cooking patterns. While the levels of firewood (26%) and induction stove (10%) ownership in the 157 households participating in this study are lower than those observed in our prior studies, we believe the exposure data of the populations to be representative.

We make several contributions to the academic literature and policy discussions on the promotion of clean cooking fuels. We assess PM_2.5_ exposure and stove use patterns in a middle-income country with a well-developed LPG market supported by decades of consumer subsidies that cover 90% of the cost of cylinder refills. Most households in our study regions pay between 2.50 and 3.50 USD for a 15-kg cylinder refill. In addition, we assess PM_2.5_ exposure among peri-urban and rural households using induction stoves, a promising but understudied clean cooking option. We build on previous cooking-related studies that have shown the multiple factors that may affect personal air pollution exposure, including stove use and time-use patterns [14–20]. However, few previous studies have combined time-resolved sensor data that may be able to disentangle the impact of simultaneous use of various stoves and fuels on personal air pollution exposure. We also offer a comprehensive discussion of the successes and limitations of our deployments, including a thorough investigation of wearing compliance.

## Methods

Previously, we administered 808 energy access and use surveys in peri-urban and rural households across four provinces of Ecuador between September 2018 and January 2019. Of these households, we enrolled 160 to participate in this study (*N* = 2 households were lost due to data collection errors). Sample size was determined by time and budget, given the exploratory nature of this study. Data collection took place between January and March 2019 in Alajuela, Manabí (rural), San Mateo, Esmeraldas (peri-urban), La Merced, Pichincha (rural), and Cayambe, Pichincha (rural) (Fig. S1). All analyses in this study were carried using R version 3.6.0, with code available upon request [21].

### Participant recruitment

The study team approached local leaders to invite participating families and representatives from other households to a discussion of the present study to familiarize participants with field workers and project goals. Local guides aided in-field logistics and helped build trust with participating households. Participants were primary cooks over 18 years of age who were available for the 48-h monitoring period.

### Estimating personal exposure to fine particulate matter

We deployed two lightweight, wearable sensors to estimate the primary cook’s personal exposure to PM_2.5_ during the 48-h monitoring period. All study participants wore the PATS+ (EME Systems; Berkeley, CA, USA) affixed to a vest at the shoulder and a 20% sub-sample also wore a co-located Ultrasonic Personal Aerosol Sampler (UPAS; Access Sensor Technologies; Fort Collins, CO, USA) (configuration shown in Fig. S2). In addition, 10% of households wore two PATS+ devices for intra-device calibration; the two co-located PATS+ estimates were averaged in analysis. The PATS+ is a light-scattering device that records time-resolved PM_2.5_ concentrations and has a three-axis accelerometer that provides a binary measure of motion for each data point recorded [22].

The UPAS device is a gravimetric filter-based instrument for estimating personal PM_2.5_ exposure [23]. Filters were weighed before and after deployment at Columbia University using methods established in previous studies [24–26]. We deployed two “field blanks” per study area (*N* = 8). Field blanks were treated as all other filters, including being taken out of their cassette and loaded into the UPAS, but with no air drawn through the device. The average difference between pre- and post-weights for field blanks (Mean ± SD: 0.0046 ± 0.0014 g) was subtracted from all filter weights (Mean ± SD: 0.0600 ± 0.0367 g) prior to analysis. In doing so, we aim to estimate the amount of contamination due to filter handling, rather than from air pollution exposure. The PATS+ and UPAS have been laboratory validated and field tested in similar studies previously [22, 27–30].

Optical sensors for estimating PM_2.5_ concentrations can be subject to bias due to a variety of factors, including varying particle size distributions, particle composition, and high-frequency vibrations of the device [31]. We co-located UPAS devices to correct PATS+ estimates in a three-step process. First, where devices had been co-located, we divided the mean PM_2.5_ concentration estimated from the PATS+ by the paired UPAS-derived estimate over the same time period. Fig. S3 compares estimates from co-located devices in a scatter plot and a Bland–Altman plot. On average, the PATS+ overestimated mean 48-h PM_2.5_ exposure by 9.7 μg/m^3^ (95% CI: 0.5–19.0 μg/m^3^) as compared with the UPAS-derived estimates. Second, we averaged all correction factors within a community, estimating a community-level correction factor (Mean ± SD: 1.25 ± 0.07). Finally, we divided all PATS+ estimates by the community-level correction factor to obtain final personal PM_2.5_ exposure estimates. Community-level correction factors improved agreement between the PATS+ and UPAS estimates (Fig. S4).

We used the UPAS-derived estimate for 48-h PM_2.5_ exposure for one household with PATS+ information lost due to data entry error.

### Capturing the use of stoves and fuels

We deployed three types of stove use monitors (SUMs) to accommodate LPG, traditional wood-burning, and induction stoves. First, we deployed iButton temperature data loggers (Maxim Integrated DS1921G; San Jose, CA, USA) in the corner of each LPG burner (Fig. S5). These commonly-used coin-sized devices record temperature every 120 s [22, 32]. Second, since cooking on firewood stoves reaches temperatures in excess of the iButton’s maximum threshold of 85 °C, we deployed thermocouple data loggers (Lascar EL-USB-TC; Whiteparish, Wiltshire, United Kingdom) that extend a heat-resistant probe (rated up to 482 °C) to the combustion zone from a data logger and record temperature every 120 s. Third, since induction stoves do not have a traditional heating element, we utilized current-voltage loggers that record changes in the current passing through the power cord every 60 s (Supco LOGiT LCV; Allenwood, NJ, USA).

We additionally piloted the use of a thermocouple system with extended battery life and data storage capacity in eight households for five continuous months on firewood-burning stoves (Geocene Dots; Vallejo, CA, USA).

#### Identifying cooking events

Our strategy for identifying cooking events primarily seeks to capture active combustion. Stove use was estimated using SUMSarizer (Version 2.0), an open-source R package developed to detect stove use from temperature time series data [33]. Using the “fire finder” function, stove use was estimated using a multi-step algorithm based on changes in temperature. First, we labeled all temperatures greater than 38 °C as cooking events—a threshold rarely exceeded without a cooking-specific steep temperature increase in our study sample. Long runs (100 data points or either 100 or 200 min depending on device sampling frequency) with negative slopes were marked as not cooking. Times with highly positive slopes (80% of temperature differences at least a doubling in temperature over 100 data points) were marked as cooking and those with highly negative slopes are marked not cooking. To improve continuity, we established a minimum length of time for “not cooking” events (30 min) and a minimum length of time for cooking events (5 min).

After using the fire finder function, firewood stove data were reviewed visually. Modifications based on visual inspection were few and fell into two groups: (1) The removal of short cooking events erroneously defined due to increases in temperature at the beginning or end of the time series, potentially due to handling of the thermocouple, and (2) Event coding for files where temperature values were offset tens of degrees negatively due to programming errors during device launch.

### Assessing participant location

We placed a GPS watch (Suunto Ambit3 Vertical; Vantaa, Finland) into a hidden inside pocket of the participant’s monitoring vest. Points within a 30 m geofence of the home’s centroid were marked as “in the home.” Larger geofences were considered, but not utilized because they appeared to capture activities outside the house (Fig. S6).

We placed a Bluetooth receiver (Berkeley Air Bluetooth Beacon Logger; Berkeley, CA, USA) near one of the participant’s stove in 20% of households (the same as those receiving the UPAS device). If the participant had multiple stoves, we prioritized placement based on research interest: (1) firewood, (2) induction, and (3) LPG. A Bluetooth beacon in the monitoring vest generated signal when near the beacon. We estimated participants’ presence “in the kitchen” based on the RSSI signal strength, which is a continuous number that ranged between −40 and −100 in our study, where −40 was the beacon and receiver touching and −100 was ~50 feet with no interferences like walls or people.

Although Bluetooth beacons have been increasingly utilized in cooking-related studies in recent years [34–38], there is no universal method for estimating distance using signal strength due to device- and environment-specific variabilities. Resource constraints did not allow for in-field calibration that can aid in classifying signal strength into proximity zones [34]. We processed signals with statistical and smoothing algorithms to remove signal noise and capture deviations due to participant’s movement, as in other contexts (described in Supporting Information).

### Analytical approach

All data from the multiple sensors were integrated to better understand the associations between PM_2.5_ exposure and cooking fuel. We aggregated 60-s sensor data to 120 s intervals to synchronize with SUMs data.

#### Fine particulate matter exposure

We characterize the distribution of 48-h average PM_2.5_ exposures by community and then 10-min averages by hour of day. Next, we discuss differences in mean 48-h exposures and 10-min average exposures by primary cooking fuel (LPG, firewood, or induction) and by ownership of a firewood stove. The distribution of exposure—including peaks—is valuable for understanding the nature of exposures (e.g., types of sources) and health risks. We use Kolmogorov–Smirnov tests to assess whether the empirical distribution functions of exposures are different by fuel stack.

#### Stove use

We characterize the timing of stove use by hour of the day (percent of all measurements during this time showing a stove is in use) and day of the week (percent of all households with measurements from the given day of the week showing use of the stove). We then estimate total stove use during the 48-h monitoring period and the duration of cooking events by stove type. Finally, we use long-term SUMs monitoring of firewood stoves to assess cooking patterns over several months and compare to observed use in the 48-h periods.

#### Attributing exposure to contemporaneous events

We carried out a series of mixed effects regression models to attribute changes in personal PM_2.5_ exposure to contemporaneous conditions like detected stove use and participant location. We utilized 10-min averaged PM_2.5_ exposure as the outcome of interest and binary explanatory variables—LPG stove in use, firewood stove in use, induction stove in use, participant wearing the vest (PATS+ motion sensor), participant in the household (GPS sensor), and participant in the kitchen (Bluetooth beacon)—in simple linear regressions with dummy variables for participant to account for within-subject clustering over time (Eq. 1). Here, we utilize only data from daytime hours (6 am–10 pm) to improve attribution between events and exposure by removing consistently low nighttime exposures (at or below the limit of detectability of around 10 μg/m^3^).

1 $${\mathrm{Average}}10{\mathrm{minute}}\,{\mathrm{PM}}_{2.5}{\mathrm{exposure}}_{i,j} = \, 	\beta _0 + \beta _1 \times Event\,Occurs_{i,j} \\ 	 + \beta _2 \times Participant\,ID_i + \varepsilon _{i,j}$$

In Eq. 1, the outcome is average 10-min PM_2.5_ exposure for participant *i* and observation *j* (every 2 min), β_0_ represents the intercept, β_1_ is the coefficient of interest (the mean difference in 10-min PM_2.5_ exposure associated with an event occurring as compared with not occurring), *Event Occurs*_*i,j*_ is a binary variable indicating if the event of interest occurred in the prior 10 min *j* for participant *i*, β_2_ is the coefficient associated with each *Participant ID*_*i*_ dummy variable that control for within-subject clustering over time, and *ε*_*i,j*_ is the error term associated with each observation for each participant.

We expand on these single-event models by assessing the co-occurrence of LPG and firewood cooking events and including an interaction term (Eq. 2). To best assess the contribution of cooking events to exposure, we further limited daytime hours’ measurements to observations where motion was detected in the prior 10 min (the temporal unit of interest).

2 $${\mathrm{Average}}\,10\,{\mathrm{minute}}\,{\mathrm{PM}}_{2.5}{\mathrm{exposure}}_{i,j} = \, 	\beta _0 + \beta _1 \times LPG\,Use_{i,j} \\ 	 + \beta _2 \times Firewood\,Use_{i,j} \\ 	 + \beta _3 \times LPG\,Use_{i,j} \times Firewood\,Use_{i,j} \\ 	 + \beta _4 \times Participant\,ID_i + \varepsilon _{i,j}$$

As in Eq. 1, the outcome in Eq. 2 is average 10-min PM_2.5_ exposure for participant *i* and observation *j* (every 2 min). In Eq. 2, rather than a single variable for an event occurring, we include binary variables for *LPG Use*_*j*_ and *Firewood Use*_*j*_ and an interaction term for the two for each observation *j* for each participant *i* to account for the co-occurrence of cooking events. Eq. 2 controls for within-subject clustering using dummy variables for *Participant ID*_*i*_ and error associated with each observation *ε*_*i,j*_ as in Eq. 1. By including an interaction term, we can estimate the association between LPG stove use alone, firewood stove use alone, and simultaneous use of LPG and firewood stoves.

We note that while our primary definition of cooking events from stove use monitors most directly seeks to capture active combustion, in attributing exposure to contemporaneous events we utilize any event that occurred in the prior 10 min. We intend to capture non-cooking moments that still emit relevant pollutants while avoiding erroneously including substantial non-cooking, non-emitting moments by using a moving 10-min window. It is plausible that we underestimate the contribution of cooking events to exposure as a result of this approach. Fig. S10 shares temperature time series and cooking event detection data from five firewood stoves, demonstrating the extent to which our stove use detection criteria captures stove die-off for firewood stoves.

### Estimating wearing compliance and personal air pollution exposure

We computed a binary variable of whether the device had been worn in the prior 10 min. We then investigated the association between compliance and personal PM_2.5_ exposure using scatterplots, regression models, and by comparing subsets of higher and lower compliance among 48-h and 24-h averages (methods described in full in the Supporting Information). Given the similarity between these two datasets (presented in “Wearing compliance and personal PM_2.5_ exposure” section), our main results utilize the full data.

### Ethical considerations

Survey data were collected and managed using REDCap software hosted at Universidad San Francisco de Quito^38^. This study was reviewed and approved prior to initiation of the research by the Institutional Review Boards at the Columbia University Medical Center and the Bio-Ethics Committee at the Universidad de San Francisco de Quito. All participants provided informed consent using REDCap’s e-signature feature. Paper copies with investigator contact information were left with participants. Participants were compensated with dish towels (~5 USD in value) after the monitoring period.

## Results

All but five of 158 participants had an LPG stove. LPG was the primary cooking fuel for 92% of households (Table 1). In comparison, 41 (26%) households owned a traditional firewood stove and 17 (10%) households had an induction stove. These stoves were rarely the household’s primary cooking option (Firewood: *N* = 8; Induction: *N* = 9). Most households with traditional firewood stoves used them in outdoor kitchens—often only enclosed on one or two sides—adjacent to the household (representative photograph shown in Fig. S7). Almost all primary cooks were women (95%) and most were between 33 and 54 years of age. Households had on average 4.8 ± 2.4 members.

**Table 1:** Characteristics of the participants and study households.

	Mean (SD) or *N* (%)	Range	Observations
Primary cook characteristics			
Age, Mean (SD)	44.37 (15.50)	18–84	158
Household Position, *N* (%)			158
Head of household	72 (46%)		
Partner of head of household	64 (41%)		
Parent of head of household	11 (7%)		
Child of head of household	7 (4%)		
Other	4 (3%)		
Education level completed, *N* (%)			158
No formal primary education completed	33 (21%)		
Primary	45 (28%)		
Part of secondary	22 (14%)		
Secondary	46 (29%)		
Greater than secondary	12 (8%)		
Civil Status, *N* (%)			158
Single	43 (27%)		
Married	97 (61%)		
Divorced	6 (4%)		
Widowed	12 8%)		
Literate, *N* (%)	131 (83%)		157
Household characteristics			
Household size, Mean (SD)			158
Adults (>17 years)	2.97 (1.62)	1–10	
Children (5–17 years)	1.41 (1.39)	0–8	
Infants (<5 years)	0.47 (0.63)	0–3	
Household head education			158
No formal education completed	30 (19%)		
Primary	66 (42%)		
Part of secondary	20 (13%)		
Secondary	29 (18%)		
Greater than secondary	13 (8%)		
Household Decision Maker, N (%)			158
Woman	37 (21.0%)		
Man	18 (10.2%)		
Joint Woman and Man	103 (61.8%)		
Stoves Owned, *N* (%)			158
Gas	153 (96%)		
Induction	17 (10%)		
Firewood	41 (26%)		
Meals Cooked per day, Mean (SD)	2.89 (0.59)	1–6	158
Daily Cooking Time, *N* (%)			157
<1 h	8 (5%)		
1–2 h	41 (26%)		
2–3 h	82 (52%)		
>3 h	26 (16%)		

### Deployment summary: successes and limitations

We obtained 157 48-h PM_2.5_ exposure estimates from the PATS+, co-located successfully with the UPAS in 33 households (Table 2). Most PATS+ deployments reached 48-h of runtime, with few gaps in data. However, only 40% of UPAS devices reached 48-h due to unexpectedly insufficient battery life.

**Table 2:** Summarizing sensor deployments and data yielded.

Instrument	Objective	Outcomes	*N*	Deployments	Run Time Median (IQR)	Sample Frequency	Expected data obtained^a^	Deployments yielding >90% expected data
*Personal PM2.5 exposure*								
PATS+	Measure personal PM_2.5_ exposure	Mean 48-h PM_2.5_ exposure; 10-min rolling mean PM_2.5_ exposure	157	172	49.2 h (48.8–50.0)	60 s	98.6%	97.1%
UPAS	Measure personal PM_2.5_ exposure	Mean 48-h PM_2.5_ exposure	33	33	40.5 h (32.1–47.2)	Time integrated	80.4%^a^	39.4%^c^
*Stove use monitoring*								
iButton	Measure LPG stove use	Minutes of LPG stove use per 48-h; Cooking events; Duration of cooking events	153	572	51.8 h (49.4–56.0)	120 s	99.9%	99.8%
Lascar thermocouple	Measure traditional firewood stove use	Minutes of firewood stove use per 48-h; Cooking events; Duration of cooking events	40	40	50.5 h (49.4–51.9)	60 s	100%	100%
Current voltage meter	Measure induction stove use	Minutes of induction stove use per 48-h; Cooking events; Duration of cooking events	16	16	49.2 h (48.7–50.5)	60 s	100%	100%
Geocene Dots	Measure traditional firewood stove use	Minutes of firewood stove use per week; Cooking events; Duration of cooking events	8	8	144 days (143–147)	120 s	100%	100%
*Participant location*								
GPS watch	Monitor participant location	Latitude and longitude coordinates; At home events	157	157	48.3 h (26.1–49.1)	120 s^d^	10.7%	0.6%
Bluetooth beacon	Monitor participant proximity to primary stove	Proximity to stove; Participant in the kitchen events; At home events	33	33	49.2 h (48.4–50.2)	20 s	100%	100%

^a^Computed as the total number of valid data points obtained divided by the run time for each deployment multiplied by the sampling frequency (expected data).

^b^Represents the percent of expected runtime.

^c^Percent of deployments resulting in >90% of expected runtime.

^d^Sampling frequency was variable during deployments (Median (IQR) seconds between data points = 480 (240–1560)).

Stove use monitor deployments achieved expected run times and high data completeness (Table 2). We obtained 575 iButton 48-h time series from LPG stoves. Fig. S8 summarizes the correlations between individual iButtons placed on different burners of the same stove, ranging between 0.31 and 0.38. Fig. S9 displays a time series of co-deployed iButton stove use monitors from a representative household. These figures illustrate the potential for stove use events to be missed if only one burner is monitored. In this study, deploying one iButton per stove may have missed between 35 and 45% of cooking events. In addition, we obtained 40 complete 48-h temperature time series from firewood stoves, five months of continuous data from 8 Geocene Dots from firewood stoves, and data from 16 households’ induction stoves.

GPS watches recorded only 10% of the number of data points anticipated and only one deployment obtained more than 90% of expected data (Table 2). Deployments were limited both by shorter-than-expected run times (issues with battery) and long, variable gaps between data points. Data points also had error when participants were inside households, “jumping” up to 10 m between measurements. Bluetooth beacons successfully recorded consistent data as anticipated; however, signal noise was ubiquitous leading to difficulty in interpreting data. We report these data as a cautionary note for future research that rely on similar approaches.

### Personal air pollution exposure

**Fig. 1:**
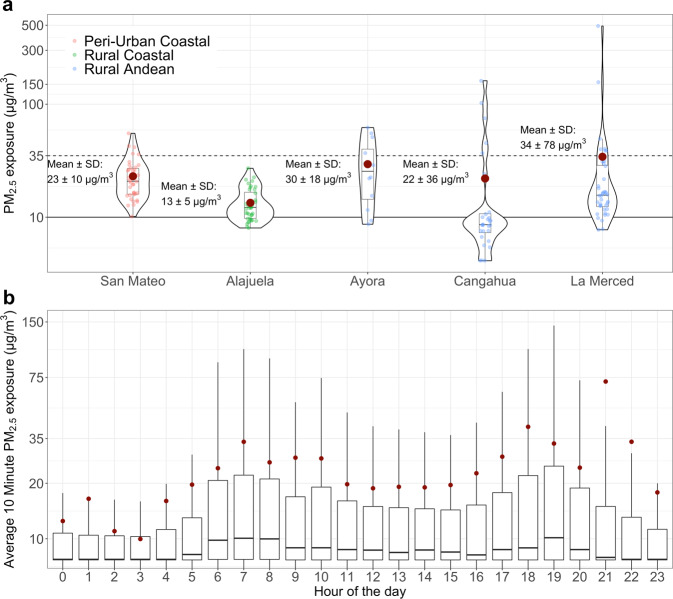
Distribution of personal PM_2.5_ exposures. **a** The distribution of average personal PM_2.5_ exposure during 48-h monitoring periods, by study community (Alajuela *N* = 40; San Mateo *N*= 38; Ayora *N* = 12; Cangahua *N* = 28; and La Merced *N* = 40). Partially transparent points show each mean personal 48-h PM_2.5_ exposure estimate within a study community, with violin plots showing the density distribution of estimates, boxplots showing the median and interquartile range of estimates, and red dots and labels showing community means. The WHO annual guideline is shown in a solid line and the interim-I guideline is shown in a dotted line. The minimum level of detection was variable across communities due to community-level correction factors which shifted minimums down from the PATS+ level of detection of 10 μg/m^3^. **b** Shows the distribution of average 10-min PM_2.5_ exposure within each hour of the day in boxplots (formed by the 25th percentile, 50th percentile median, and 75th percentiles) extended by whiskers (10th percentile and 90th percentile). Red dots show the mean exposure for each hour. The participant in La Merced with average 48-h PM_2.5_ exposure of 492 μg/m^3^ experienced a high short-term exposure of >10,000 μg/m^3^ in the evening, potentially due to a cooking event or burning mosquito coils (both reported to occur approximately at this time); this peak exposure also explains the elevated average exposure during 21:00–22:00 h in **b**. These data points have not been downward corrected because they are plausible.

Mean 48-h PM_2.5_ exposure for most participants was below the WHO interim-I guideline of 35 μg/m^3^ (*N* = 137; 87%). Still, there was heterogeneity in exposures within and across communities (Fig. 1). The highest 1-h mean exposures occurred in the early morning (6 am–10 am) and the evening (5 pm–8 pm), with their 75th percentile at ~20 μg/m^3^.

**Fig. 2:**
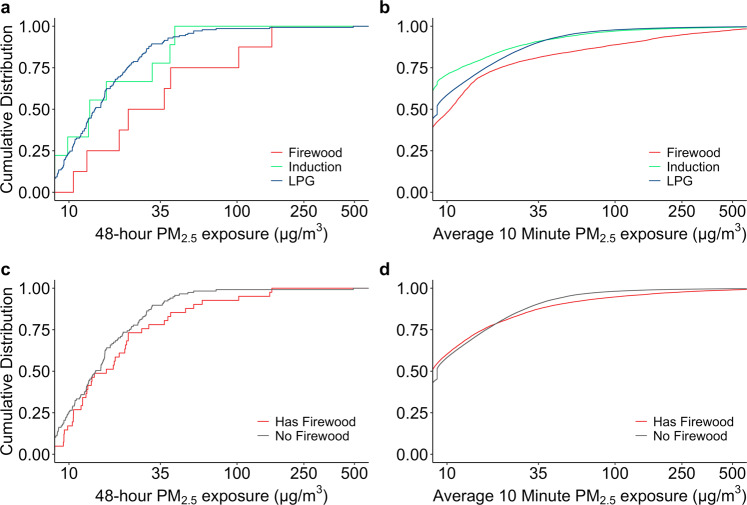
Cumulative distribution of personal PM_2.5_ exposure by cooking fuel groups. **a** Average 48-h personal PM_2.5_ exposures among primary cooking fuel groups (firewood, *N* = 8; induction, *N* = 9; LPG, *N* = 141). **b** Each 10-min moving average PM_2.5_ exposure during daytime hours (6 am–10 pm) during the 48-h period by primary cooking fuel group. **c** and **d** replicate **a** and **b** but by ownership of a traditional firewood stove (has firewood, *N* = 41; no firewood, *N* = 117).

Table S1 presents descriptive statistics for mean 48-h personal PM_2.5_ exposures for all fuel stacking combinations. Cooks primarily using induction had an average 48-h PM_2.5_ exposure of 20.3 ± 13.8 μg/m^3^, which was similar to primary LPG users (22.4 ± 43.2 μg/m^3^) (Fig. 2a). Primary firewood users had higher average 48-h PM_2.5_ exposures than primary LPG users (50.8 ± 53.4 μg/m^3^). However, only 8 participants reported using firewood to meet most of their cooking needs. Average 48-h PM_2.5_ exposure was 23.6 ± 27.5 μg/m^3^ among households that used firewood as a secondary fuel (*N* = 33; 21% of the total study sample; 80% of firewood users) (Fig. 2c). This exposure was not significantly different from exposure among cooks that did not have a firewood stove (21.9 ± 45.3 μg/m^3^; *P* = 0.32).

While ownership of a firewood stove is useful for estimating exposure based on an easily-collectible data point, assessing firewood stove use facilitates a more direct estimate of the association between solid fuel combustion and air pollution exposure. Average 48-h PM_2.5_ exposure was 40.4 ± 43.1 μg/m^3^ for households that used their firewood stoves for at least 30 min during the monitoring period (*N* = 23). Firewood stove owners not using their stoves during the monitoring period (*N* = 18) had lower exposures (14.2 ± 7.9 μg/m^3^) (Welch Two-Sample *t*-test: *P* = 0.02). Overall, Fig. S11 shows that participants using their firewood stove for more than 30 min during the 48-h monitoring period had higher distributions of average 48-h PM_2.5_ exposure and 10-min PM_2.5_ exposures than non-users (Two-Sample Kolmogorov–Smirnov Tests: *P* = 0.002; *P* < 0.001). In addition, there was a positive, but not statistically significant, association between minutes of firewood stove use and personal air pollution exposure among firewood stove owners (Fig. S12).

Primary firewood users experienced elevated short-term exposures significantly more frequently than those primarily using a clean cooking fuel (Fig. 2b). For example, primary firewood users experienced 10-min average PM_2.5_ exposures greater than 35 μg/m^3^ for 19% of the daytime monitoring period (vs. 10% for primary LPG; primary induction: 9%) and above 50 μg/m^3^ for 16% of the daytime (vs. LPG: 6%; Induction: 6%). Two-Sample Kolmogorov–Smirnov tests confirm that the overall distribution of 10-min exposures for primary LPG users were significantly different from primary firewood users (*P* < 0.001).

Firewood stove owners and non-firewood-stove owners had more similar distributions of short-term PM_2.5_ exposure (Fig. 2d). Still, households with a firewood stove experienced 10-min average exposures greater than 35 μg/m^3^ somewhat more frequently than those without a firewood stove (10% compared with 6%).

### Stoves used during the monitoring period

**Fig. 3:**
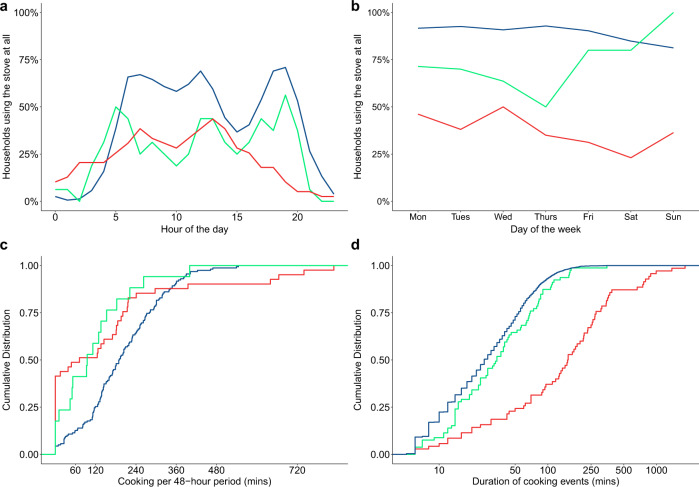
Characterizing detected stove use by hour of the day, day of the week, and total minutes used during the study period. **a** Lines connect estimates of the fraction of minutes during each hour that the designated stove was in use throughout the monitoring period. **b** Lines connect estimates of the fraction of minutes in each day of the week where the stove was in use. **c** Cumulative distribution function showing the fraction of participants with the overall number of detected minutes of cooking during the 48-h monitoring period. **d** Cumulative distribution function showing the distribution of the duration of discrete cooking events by fuel type. Axis has been log-transformed due to clustering at low values.

LPG stoves were used throughout the day and each day of the week by most households (Fig. 3a and b). In comparison, firewood stoves were mostly used in the afternoon and evening. Among owners, firewood stoves were used by about 50% of households each day. Although induction stoves were used less overall than LPG stoves, they were used during all meal times and days of the week.

On average, households used their LPG stoves for 200 ± 116 min in the 48-h monitoring period compared 446 ± 595 min for firewood stoves and 114 ± 107 min for induction stoves. There was substantial variation in the distributions of stove use by fuel type (Fig. 3c). For example, while about half of households made limited or no use of their firewood stoves during the monitoring period, a few households used their firewood stoves much more than most households used their LPG stoves.

Firewood stove cooking events were longer than LPG or induction stove cooking events (Fig. 3d). On average, firewood cooking events were 256 ± 329 min long, though the median was somewhat lower (153 min; IQR: 61–277). The durations of induction and LPG cooking events were similar in distribution (Induction median (IQR): 34 min (15–69); LPG median (IQR): 26 min (12–54)).

Long-term firewood stove monitoring (duration range: 108–210 days) shows similar use patterns to 48-h monitoring in the same households (Fig. S13). Four of the eight households showed sparing use (once per month), two showed more frequent use (once per week), and two showed zero use.

#### Multiple stove use

Figs. S14 and S15 show stove use patterns over the monitoring period for an average household with (1) both LPG and a firewood stove and (2) both LPG and an induction stove, respectively. These time series exemplify two modes of fuel stacking. In the first, different stoves and fuels are used during different cooking events. In the second, multiple stoves and fuels complement each other and are used during the same cooking event.

Overall, among households stacking LPG and firewood, the LPG stove was used in 35% of hours where the firewood stove was used. Stove stacking was even more prevalent for households owning LPG and induction: LPG stoves were used in 65% of hours that an induction stove was used.

Still, LPG was often used to cook entire meals on its own in both fuel stacking scenarios. In firewood and LPG stacking scenarios, firewood was used during about 16% of hours when the LPG stove was in use. When induction and LPG were owned together, induction was used during 26% of the hours LPG was in use.

### Attributing exposure to contemporaneous events

**Fig. 4:**
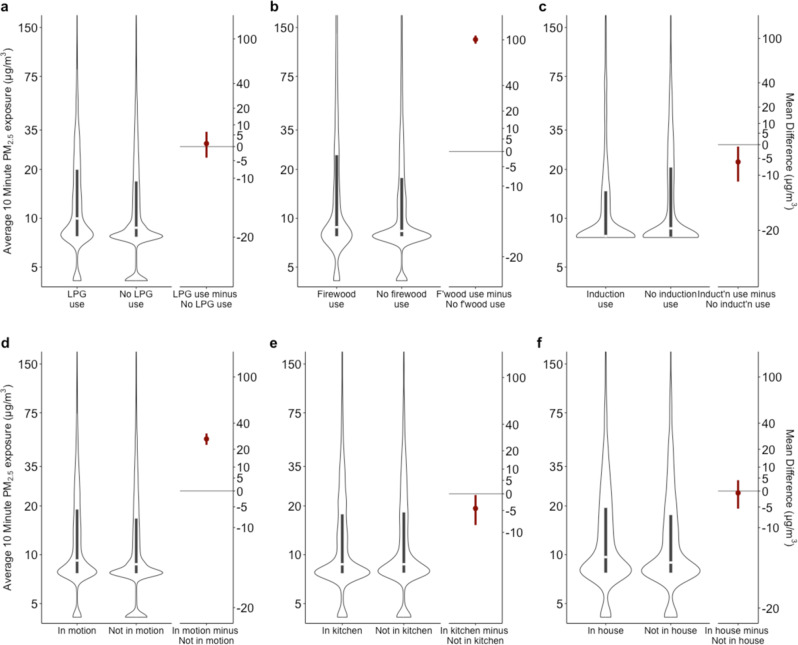
Association between contemporaneous characteristics and personal PM_2.5_ exposure during waking hours (6 am–10 pm). **a**–**f** Gardner–Altman plots designed to show the distribution and mean difference between two groups [60, 61]. The left side violin plots of the distribution of average 10-min personal PM_2.5_ exposure and boxplots (boxes show 25th percentile, 50th percentile as a white line, and 75th percentile). The right sides show the mean difference and 95% confidence intervals between the two distributions, accounting for within-subject clustering over time (Eq. 1). Both *y*-axes are logarithmic to account for the right-skewed distribution of personal exposures. Right side *y*-axes for the mean difference are centered and relabeled around 0 (mean for the event not occurring) but follow the same units as the left side. Points above 150 μg/m^3^ contribute to the plots and estimations, but are not shown. Flat bottoms on violin plots are from the lower limit of detection, which varied from community to community as described in “Estimating personal exposure to fine particulate matter” section.

Figure 4 assesses the contribution of each fuel to personal exposures by comparing the distribution of exposures when each fuel was in use to the distribution when it was not in use. Similarly, we assess the contribution of wearing compliance (wearing vs not wearing) and location (in kitchen vs. not in kitchen; at home vs. not at home). We use Eq. 1 to estimate the difference in 10-min average PM_2.5_ exposure for each comparison.

Contemporaneous firewood cooking was associated with 101 μg/m^3^ higher 10-min PM_2.5_ exposure (95% CI: 94–108 μg/m^3^ higher). LPG cooking events were not associated with 10-min PM_2.5_ exposure (1.2 μg/m^3^ higher; 95% CI: 4.0 μg/m^3^ lower–6.5 μg/m^3^ higher). Induction cooking events were not associated with 10-min PM_2.5_ exposure (4.4 μg/m^3^ lower, 95% CI: 10.1 μg/m^3^ lower—1.3 μg/m^3^ higher).

When the PATS+ recorded that the device was in motion—when we assume that it was being worn and capturing personal exposure most effectively—10-min PM_2.5_ exposure was 27.4 μg/m^3^ higher (95% CI: 22.9–31.9 μg/m^3^). Exposure was not different when the GPS watch location suggested that the participant was home. Somewhat surprisingly given the expectation that kitchen area concentrations would be higher than elsewhere, 10-min PM_2.5_ exposure was not associated with the participating being in the kitchen (3.1 μg/m^3^ lower, 95% CI: 7.6 lower—1.4 μg/m^3^ higher).

#### The contribution of concurrent firewood and LPG cooking events to exposure

The preceding analysis does not account for simultaneous use of multiple fuels. In our data, however, households often used multiple stoves during a single meal. We estimate 121 μg/m^3^ higher 10-min average PM_2.5_ exposure (95% CI: 97–145 μg/m^3^) when the firewood stove was in use but the LPG stove was not. When both stoves are in use, we estimate a somewhat smaller increase in exposure (74 μg/m^3^; 95% CI: 52–141 μg/m^3^) (Fig. S16). LPG stove use on its own was not significantly associated with increased 10-min average PM_2.5_ exposure in this model (2.3 μg/m^3^ lower; 95% CI: 19 μg/m^3^ lower—23 μg/m^3^ higher).

### Wearing compliance and personal PM_2.5_ exposure

Using the PATS+ accelerometer-derived measure of motion, we estimate that participants wore their vests for 12% of daytime hours. Wearing peaked during the middle of the day and again in the evening (Fig. S17), suggesting that participants wore their vests particularly for cooking meals. Compliance was significantly positively correlated with detected LPG stove use (*r* = 0.24), induction stove use (*r* = 0.22), and firewood stove use (*r* = 0.14) (Fig. S18). Furthermore, participants wore their vests for 40% of observations with detected cooking events. Compliance did not vary significantly based on primary cooking fuel, firewood stove ownership, or induction stove ownership (Fig. S19).

We assessed the association between wearing compliance and personal PM_2.5_ exposure in three data subsets (Fig. S20). We observed no association between average 48-h wearing compliance and PM_2.5_ exposure or between average daytime wearing compliance and daytime PM_2.5_ exposure. In addition, there was no significant association between wearing compliance and PM_2.5_ exposure when the full monitoring period was divided into two daytime averages. Accounting for within-subject clustering, in this 24-h daytime subset, we observe no significant association between a 10% increase in wearing compliance and mean 24-h PM_2.5_ exposure (9 μg/m^3^ higher; 95% CI: 21 μg/m^3^ lower—39 μg/m^3^ higher).

Personal PM_2.5_ exposure was not significantly different across households above and below 30% daytime 24-h compliance. Those with compliance below 30% (*N* = 282 observations) had mean personal 24-h daytime PM_2.5_ exposure of 27.8 ± 80.6 μg/m^3^ (median (IQR): 15.1 μg/m^3^ (10.3–25.9 μg/m^3^)). In comparison, participants with compliance above 30% (*N* = 28 observations) had mean personal 24-h daytime PM_2.5_ exposure of 28.9 ± 28.4 μg/m^3^ (median (IQR): 17.4 μg/m^3^ (14.1–30.1 μg/m^3^)).

Fig. S21 shows similar distributions of average 48-h PM_2.5_ exposure by common cooking fuel use categories—(1) LPG as the primary cooking fuel and (2) ownership of a firewood stove—between a subset of participants with greater than 10% of samples detecting motion (about 6 h) and the full sample. Fig. S22 replicates these results using 24-h samples.

## Discussion

We used a diverse and comprehensive set of sensors to estimate personal PM_2.5_ exposure and assess stove use in peri-urban and rural Ecuador in a 48-h period. To our knowledge, this is the first study of its kind in Ecuador. Ecuador’s decades-long investment in LPG subsidies provides a rare opportunity to assess the PM_2.5_ exposure and risk implications of abundant, low-cost LPG.

We found that 87% of average 48-h personal PM_2.5_ exposures fell below the WHO interim-I guideline (35 μg/m^3^), though only 23% are below the full air quality guideline (10 μg/m^3^) [39]. Nevertheless, persistent firewood use continues to affect PM_2.5_ exposure. The distributions of average and short-term personal PM_2.5_ exposures were significantly higher among households using firewood stoves than those that exclusively used clean cooking fuels, but few households exceeded the WHO Interim-I standard.

Average personal PM_2.5_ exposure among primary cooks using LPG as their primary cooking fuel and firewood as a secondary option was 24 ± 27 μg/m^3^. Over the 48-h period we estimate that these primary cooks experienced short-term exposures above 35 μg/m^3^ for an average of 8.6 h (18% of the time). Furthermore, by aligning time-resolved data from stove use monitors and personal PM_2.5_ exposure sensors we estimate that cooking with firewood leads to an increase of between 100 and 120 μg/m^3^ in 10-min average PM_2.5_ exposure. Continued firewood stove use and long firewood cooking events may significantly contribute to high PM_2.5_ exposures.

We observed that firewood stoves were used predominately for lunch and dinner and that cooking events using firewood stoves were longer than LPG or induction stove cooking events. These results can likely be explained in two not mutually exclusive ways. First, firewood stoves have a significantly longer starting period of time where the fire must build compared with the instantaneous heating of LPG and induction stoves. In addition, the fire may persist beyond the cooking period. Second, firewood stoves are used for more energy-intensive meals. These results have been suggested elsewhere [40–43], including in our own questionnaire-based results [13]. Understanding motivations for continued firewood use and its attendant health risks is valuable for promoting the exclusive use of clean cooking fuels.

### Limitations

Personal PM_2.5_ exposure is the gold standard for estimating health risks from air pollution because it integrates exposures over time and space [17]. However, accurately estimating personal PM_2.5_ exposure relies on precise instruments and consistent wearing compliance [44, 45]. Our use of co-located UPAS devices to correct PATS+ measurements is a common strategy used in similar studies [46–48]; however, recent research has suggested that this approach is subject to variability in correction factors across deployments [49].

In addition, low wearing compliance can bias estimates of personal exposure [50], though the direction of the bias may depend on context. Compliance has been empirically assessed in previous studies using accelerometers [18, 19, 51–55], GPS loggers [18], pedometers [56], and wearable cameras [18, 51]. In many cases, a quantitative threshold is applied and data that do not meet the threshold are removed [18, 19, 56]. In others, compliance is not analyzed or methods for assessment are unclear [51, 54]. Raw compliance data and its association with personal air pollution exposure has rarely been presented in full [44, 45], as we do here.

We observed similarities in data between our “high” wearing compliance data (>30% daytime wearing) and across fuel types. Still, low levels of wearing compliance are a limitation and suggest that our results presented may underestimate mean personal PM_2.5_ exposure because unworn monitors might miss cooking events, which we believe were the primary reasons for high peak exposures. We aimed to achieve high wearing compliance through announced but unscheduled drop-ins with study participants during the monitoring period, group- and individual-level explanations of the importance of compliance for the study, and the commitment to return after data analysis to present and explain group- and individual-level results. Future studies might consider additional strategies for increasing compliance, perhaps including incentives.

In addition, the study’s cross-sectional study design has limited capacity to estimate annual air pollution exposure due to potential variations during the year. Such seasonality could be due to differences in fuel use patterns, meteorology, or behavioral shifts.

Our study design and sensor deployment strategy were consistent with the goal of attributing exposure to cooking events. However, while a strength of this study is the implementation of these multiple sensors, there were some issues in deployment. Notably, the GPS watch deployments did not collect data at the expected frequency or with high precision. Further validation of GPS watches to monitor participant location in rural areas is needed. We additionally deployed Bluetooth proximity beacons in a smaller subset of households to complement the GPS watches and further validate the beacons as a strategy for attributing exposure to cooking events. While the Bluetooth beacons have aided in exposure attribution in similar contexts previously [34, 35], we did not observe a robust association between cooking events, the beacon capturing participant presence in the kitchen, and personal PM_2.5_ exposure in our deployments. Additional refinement in the methods used to deploy Bluetooth beacons and protocols for converting signals to proximity is warranted.

While an ideal study would have included ambient and kitchen area air pollution monitoring beyond personal monitoring, due to budgetary constraints, we relied on personal exposure monitoring to characterize the exposure of individual primary cooks. Still, attributing exposure to cooking events may also be enhanced by deploying air pollution exposure monitors in the kitchen and those that capture ambient air pollution. Kitchen area monitors alone usually overestimate personal exposure due to time-activity patterns [17], but in combination with time-resolved personal exposure monitors may provide insights into area concentrations resulting from cooking events [35]. Ambient air pollution monitors can establish baseline exposures and potentially capture the influence of community-level air pollution, including those from nearby cooking events [57].

A strength of this study was the inclusion of a subset of households receiving long-term firewood stove use monitoring. We observed similar cooking patterns between seven of the eight households in the long-term and 48-h monitoring periods, suggesting limited bias due to being observed (the “Hawthorne Effect”), which has been observed in similar studies [58, 59]. Future studies can further explore the benefits of long-term monitoring as a tool to confirm the validity of short-term stove use monitoring data or to extrapolate short-term monitoring to better estimate health risks.

### Conclusions

We use a holistic approach to integrate multiple sensors to assess personal PM_2.5_ exposure and stove use in a setting where low-cost LPG has been accessible for decades. Significant LPG subsidies have facilitated a nationwide transition to clean fuels dominating throughout peri-urban and rural coastal and Andean Ecuador. We observe that most participants experienced personal PM_2.5_ exposure below the health-based WHO interim-I guideline of 35 μg/m^3^. Still, our results suggest that secondary firewood use is associated with average and short-term personal air pollution exposure above this interim-I guideline. These findings speak to the potential for substantial fuel subsidies to accelerate the transition to clean cooking fuels to lower air pollution exposure elsewhere. Future studies using multiple sensors can offer valuable insight into source contributions of PM_2.5_ and exposure risks for populations stacking cooking fuels.

## Supplementary information

Supplementary information
