# Supplementary material for: Long-standing LPG subsidies, cooking fuel stacking, and personal exposure to air pollution in rural and peri-urban Ecuador
Source: J Expo Sci Environ Epidemiol. 2020 May 15;30(4):707–20. doi: 10.1038/s41370-020-0231-5 (PMC7316622; doi:10.1038/s41370-020-0231-5)
Supplement: Supplementary file 1 — Supplementary information [file 41370_2020_231_MOESM1_ESM.docx]

**SUPPORTING INFORMATION**

**Long-standing LPG subsidies, stove use, and low personal exposure to air pollution in rural and peri-urban Ecuador**

^1^ Department of Environmental Health Science, Columbia University Mailman School of Public Health, New York, NY, USA

^2^ Independent Consultant, Quito, Ecuador

^3^ Department of Mechanical Engineering, Universidad San Francisco de Quito, Quito, Ecuador

***Corresponding Author:**

Darby W. Jack, dj2183@cumc.columbia.edu
Department of Environmental Health Science,
Columbia University Mailman School of Public Health

11^th^ Floor, 722 W 168^th^ Street, New York, New York 10032

**Funding:** The authors acknowledge support from the United States National Institute of Health (NIH) Common Fund program for Global Health supported through the Clean Cooking Implementation Science Network. In addition, CFG is supported by the United States National Institute of Environmental Health Sciences (NIEHS) T32 ES007322 and DWJ by NIEHS R01 ES024489.

1. **Methods**

| 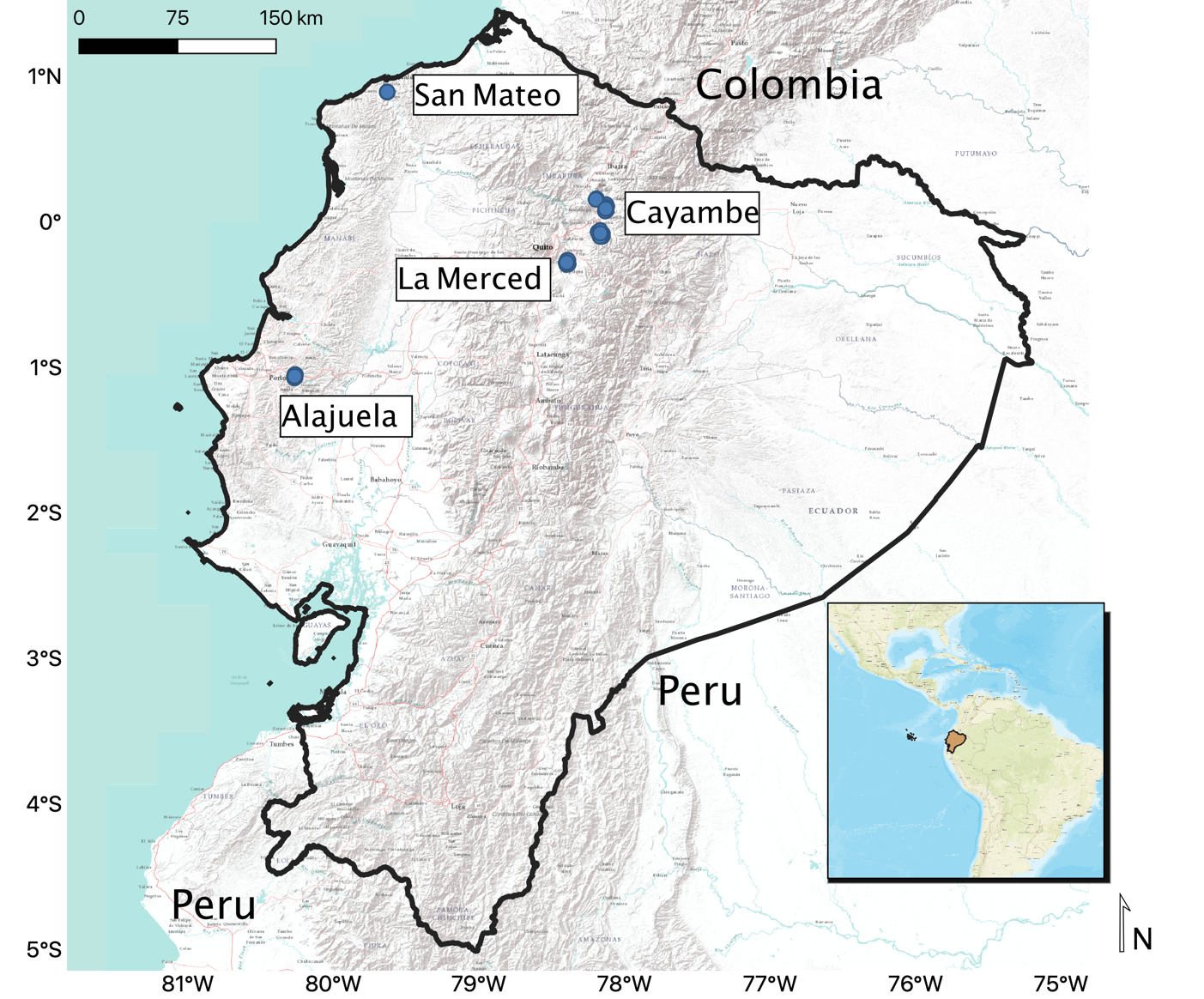 |
| --- |
| **Figure S1. Map of study communities.** |

| 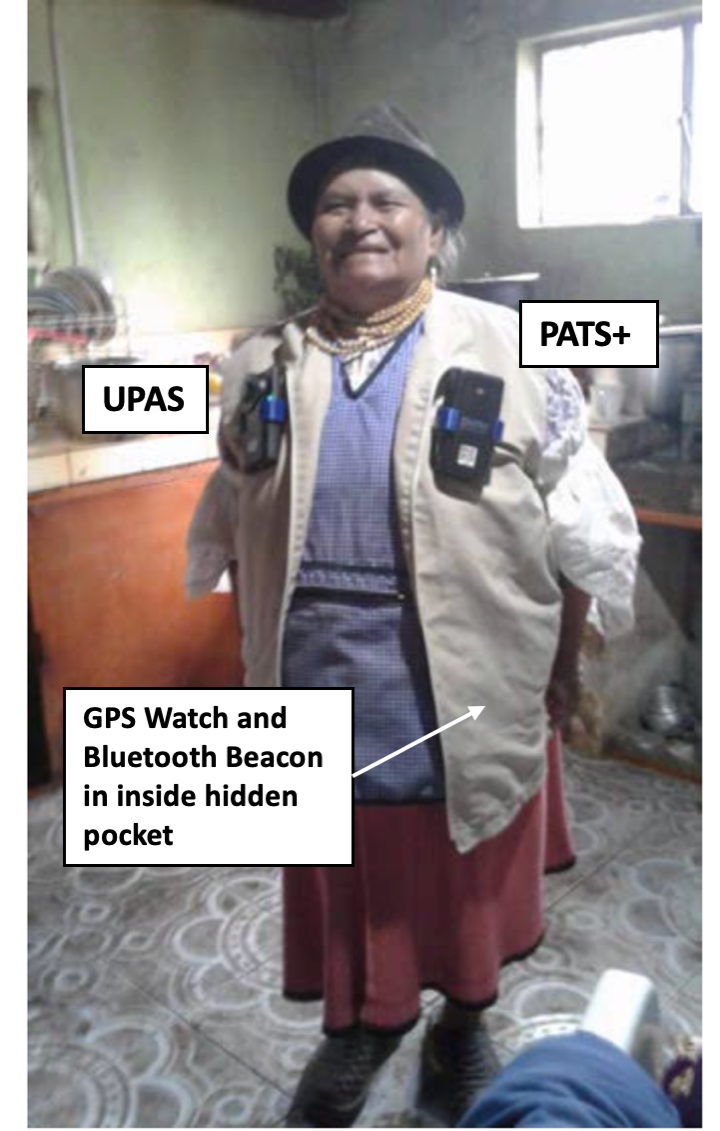 |
| --- |
| **Figure S2. Example participant monitoring vest.** The PATS+ and UPAS devices are deployed near the participant’s breathing zone and the location monitoring systems (GPS watch and Bluetooth beacon) are deployed in a hidden inside pocket. |

| 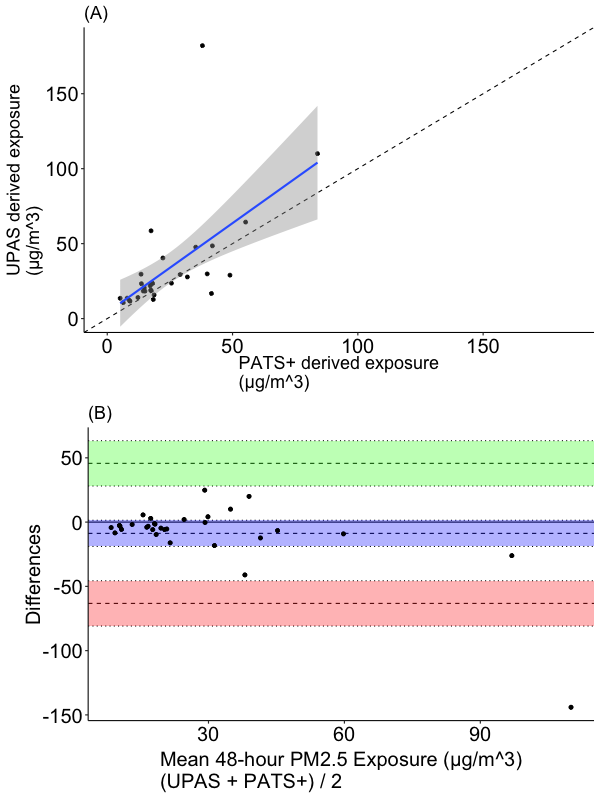 |
| --- |
| **Figure S3. Comparing UPAS and PATS+ devices.** (A) Scatter plot shown for 48-hour PM2.5 exposure estimates derived from co-located UPAS (y-axis) and PATS+ (x-axis) devices (N=40) with a linear fit model between the points (blue solid line) laid over a 1:1 line (dotted grey line). (B) Bland-Altman plot comparing 48-hour PM2.5 exposure estimates from co-located UPAS and PATS+ devices. Differences shown on the y-axis (exposure estimates from the UPAS minus exposure estimates from the PATS+) are compared with the mean exposure from the two devices. |
| 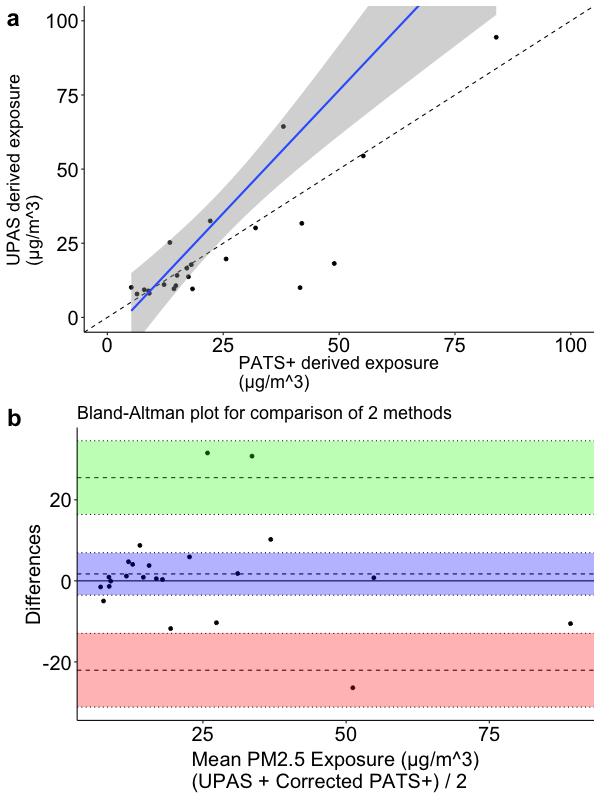 |
| **Figure S4. Comparing UPAS and corrected PATS+ deployment estimates.** (A) Scatter plot shown for 48-hour PM2.5 exposure estimates derived from co-located UPAS (y-axis) and corrected PATS+ (x-axis) devices (N=40) with a linear fit model between the points (blue solid line) laid over a 1:1 line (dotted grey line). (B) Bland-Altman plot comparing 48-hour PM2.5 exposure estimates from co-located UPAS and PATS+ devices. Differences shown on the y-axis (exposure estimates from the UPAS minus exposure estimates from the corrected PATS+) are compared with the mean exposure from the two devices. |

| 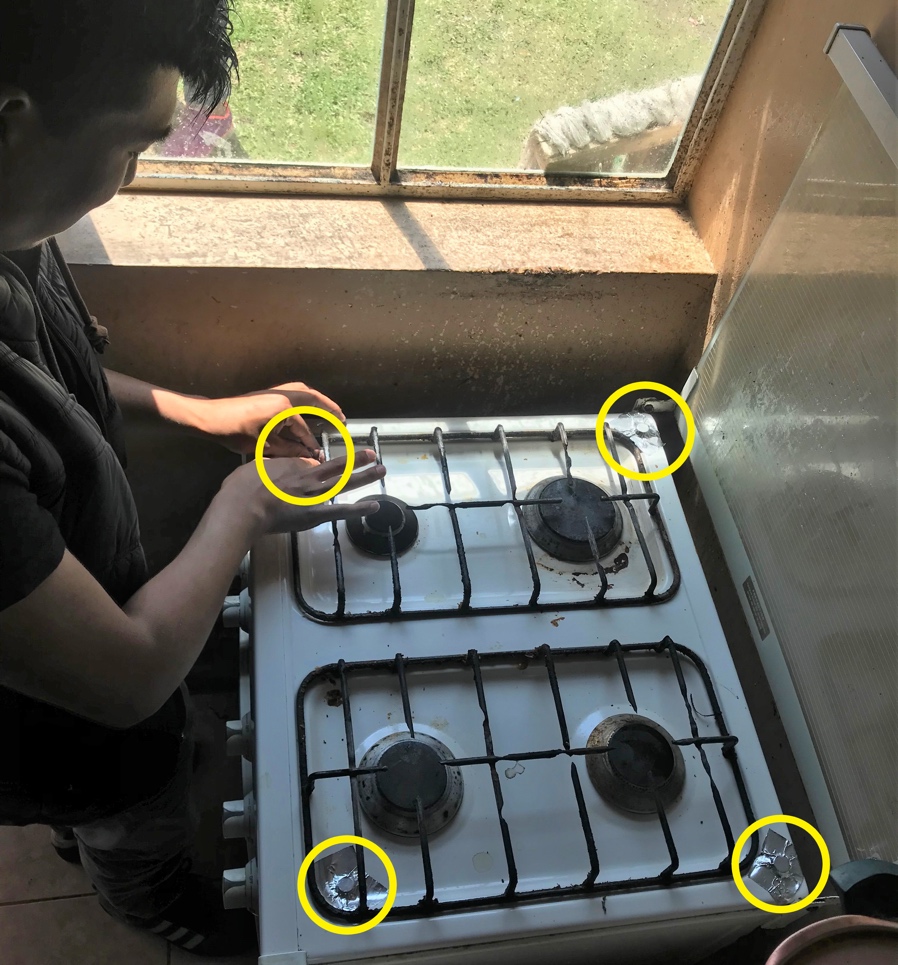 |
| --- |
| **Figure S5. Example iButton placements.** These placements were intended to ensure that all cooking events were captured, and to test the possibility that a single iButton placement might sufficiently capture cooking events in the event that the number of iButtons is constrained in future studies. |

| 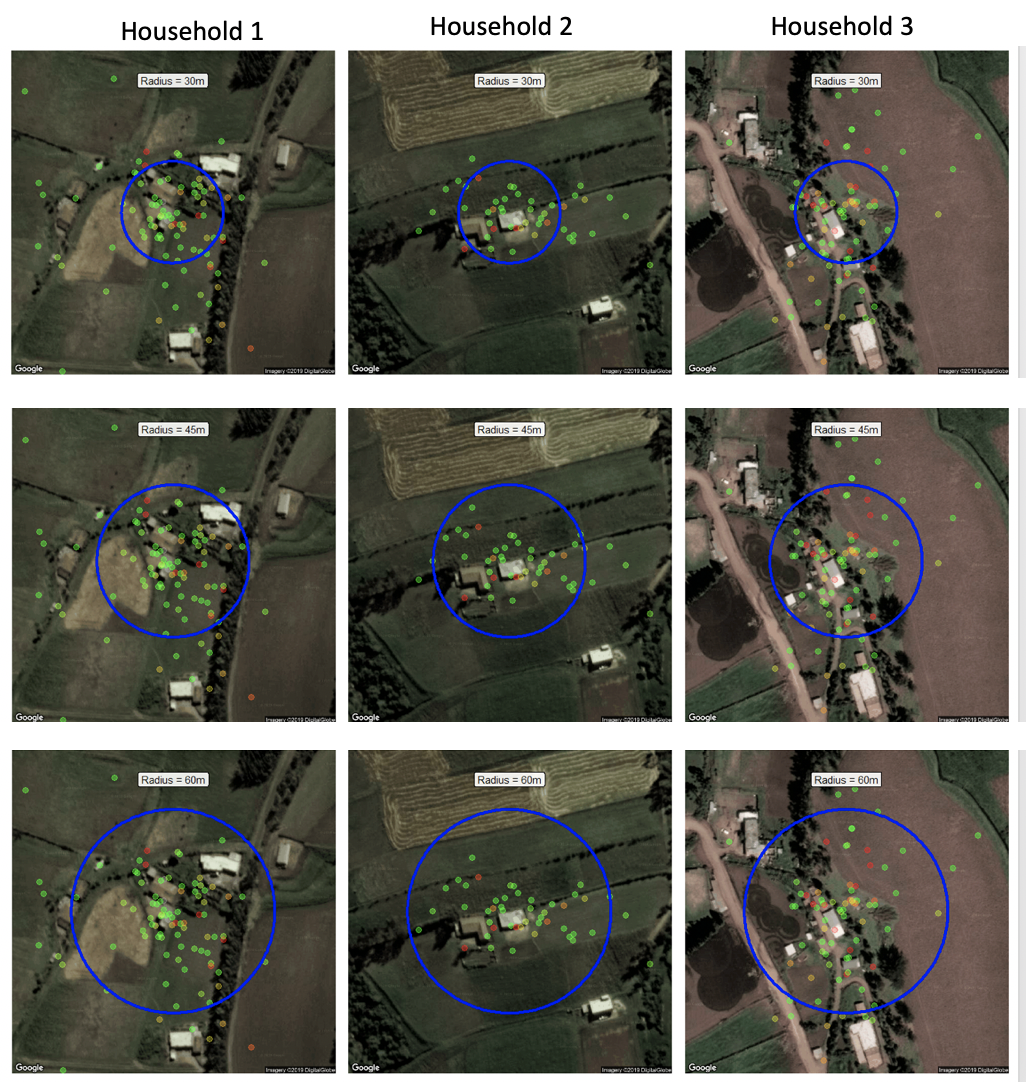 |
| --- |
| **Figure S6. Example of GPS watch data streams and geofences tested.** We used wide geofences effectively capture most points where we plausibly consider the participant to be in the household but subject to random error in the device. We chose 30 m to limit the inclusion of non-study-household areas. |

- 1. PATS+ motion sensor

**The PATS+ has a three-axis accelerometer that provides a binary measure of motion for each data point recorded. Specifically, the measure of motion is estimated by comparing the device orientation to the three-axis orientation captured 60 seconds previously using a built-in algorithm by the device manufacturer. If the device orientation has changed by more than 10 degrees on any axis then it is “in motion” and otherwise it is “not in motion.”**

- 1. Bluetooth beacon signal processing

**First, low outliers were removed by replacing them with three-minute local maximums. Then, we employed a variation of a maximum velocity filter to eliminate abrupt jumps using the signal’s exponential rate of change. Next, a three-point rectangular filter, a second maximum velocity filter, and a five-point triangular filter were used to further improve the signal. These algorithms were meant to filter out signal attenuation and noise caused by furniture, walls, human bodies, and other environmental conditions. The filtered signal was used to identify a participant’s presence “in the kitchen” when the signal was 4 units above the 80^th^ percentile of signal strength (the highest 9.6 hours of data). Short “in the kitchen” events (less than 3 minutes) and “out of the kitchen” events (less than 8 minutes) were removed.**

- 1. Wearing compliance

**We first examined when participants were wearing their vests and whether compliance coincided with cooking events. Next, we present scatterplots of average 48-hour compliance and average 48-hour PM_2.5_ exposure using daytime data. We also replicate this plot using 24-hour averages by dividing the 48-hour time series in half to increase sample size and assess any monitoring-day-specific patterns. Next, we estimate the association between 24-hour daytime wearing compliance and 24-hour daytime PM_2.5_ exposure in a linear regression, accounting for within-subject clustering over time. Finally, we present comparisons of average PM_2.5_ exposure among a subset of data with higher compliance as compared to the full sample.**

- 1. Additional notes on ethical considerations

REDCap’s e-signature feature was used to record participant’s consent after a thorough verbal explanation of the approved consent form. Paper copies of the consent form were also provided, including investigator’s contact information.

1. **Results**

| 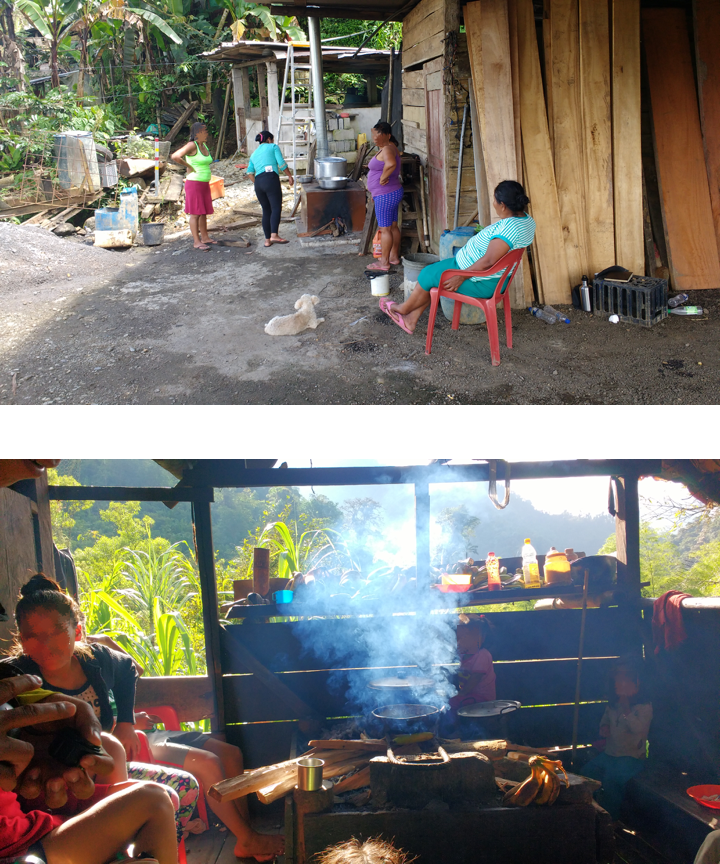 |
| --- |
| **Figure S7. Example of representative kitchens where study households used firewood stoves.** |

| 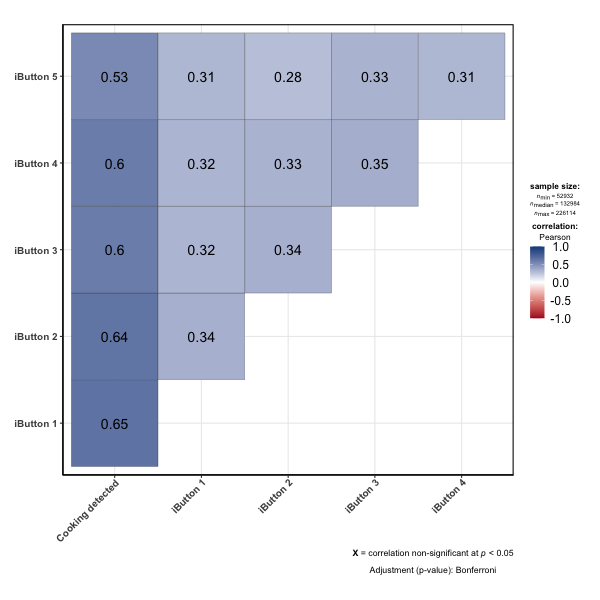 |
| --- |
| **Figure S8. Correlation between iButton sensors.** Correlation plot of estimated stove use between deployed iButton sensors on the same LPG stove during the monitoring period and detected stove use (method = Pearson; P-Value adjustment for multiple comparisons = Bonferroni). |

| 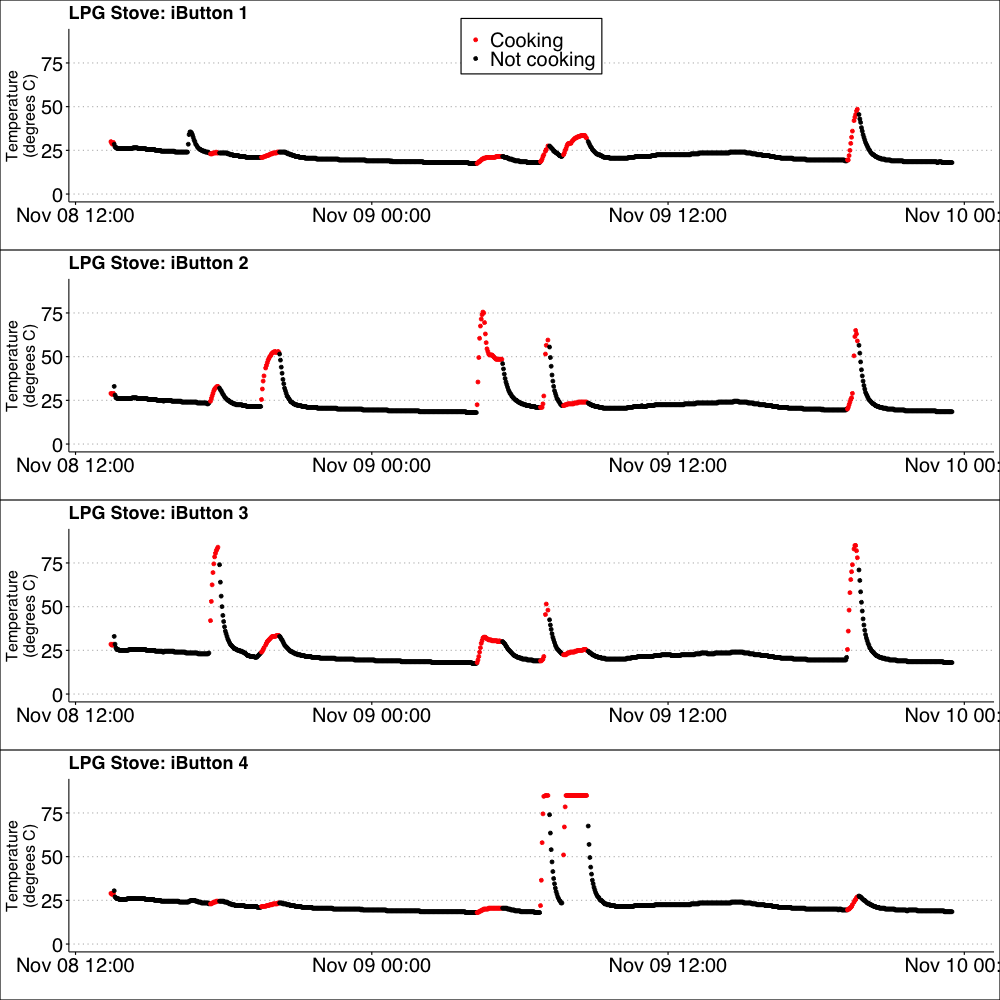 |
| --- |
| **Figure S9. Agreement between co-deployed iButton stove use monitors.** Time series data shown are from four distinct iButton devices co-deployed on the same LPG stove on different locations of the stove over the same monitoring period. Red dots indicate “detected cooking events” on the stove using all iButtons. |

| 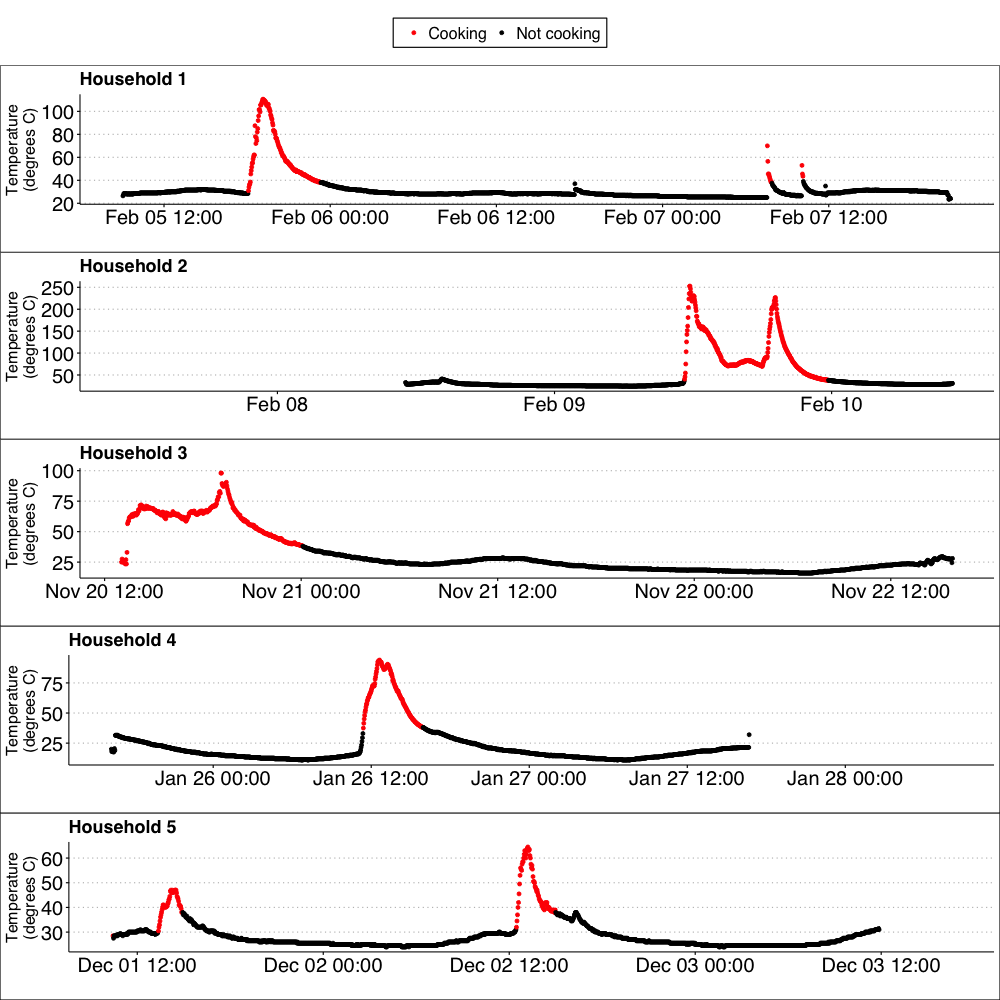 |
| --- |
| **Figure S10. Detection of cooking events on five firewood stoves (in five households).** Red dots indicate “detected cooking events.” Note that this does not include the additional 10 minutes after the final detected cooking event in a given run used in the regressions described in Section 2.5. |

**Table S1. Characterization of personal PM_2.5_ exposures for all fuel stacking combinations**

|  | **Mean (SD) ^1^** | **Median (IQR) ^1^** | **48-hour observations** |
| --- | --- | --- | --- |
| **LPG Primary** |  |  |  |
| Exclusive | 21.7 (47.9) | 14.0 (9.8, 20.7) | 104 |
| Firewood Secondary | 24.8 (28.6) | 13.9 (10.9, 22.4) | 30 |
| Induction Secondary | 22.5 (6.9) | 25.8 (18.7, 25.8) | 7 |
| Firewood and induction secondary | - | - | 0 |
| **Firewood Primary** |  |  |  |
| Exclusive | 161 (NA ^2^) | 161 (NA ^2^) | 1 |
| LPG Secondary | 34.2 (34.7) | 21.2 (14.6, 33.3) | 6 |
| Induction Secondary | 40.3 (NA ^2^) | 40.3 (NA ^2^) | 1 |
| LPG and induction secondary | - | - | 0 |
| **Induction Primary** |  |  |  |
| Exclusive | 31.7 (13.3) | 35.6 (26.7, 40.6) | 4 |
| LPG Secondary | 10.6 (3.9) | 10.6 (9.2, 11.9) | 2 |
| Firewood Secondary | 8.2 (NA ^2^) | 8.2 (NA ^2^) | 1 |
| LPG and firewood secondary | 13.3 (4.9) | 13.3 (11.5, 15.0) | 2 |

^1^ All measures displayed are concentrations in μg/m^3^.

^2^ NAs are present because only one observation is present, therefore standard deviations and interquartile ranges are not estimated or meaningful.

| 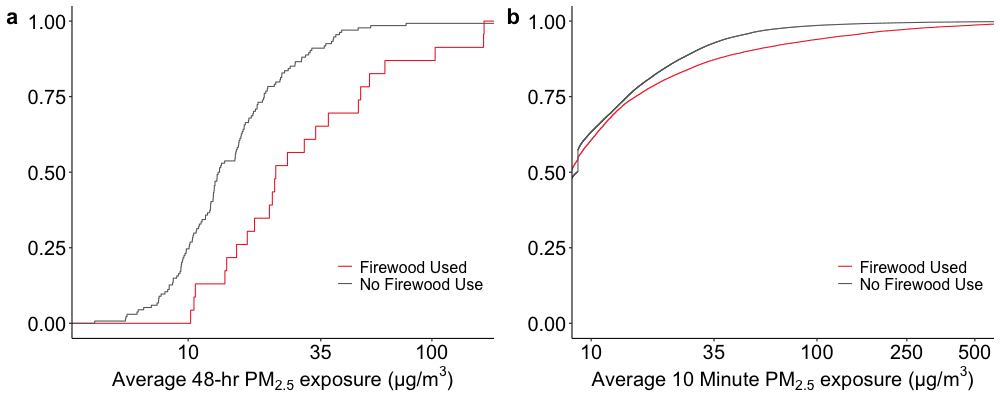 |
| --- |
| **Figure S11. Distribution of the average 48-hr PM2.5 and average 10-minute PM2.5 exposure given 30 minutes of firewood use during the monitoring period.** |

| 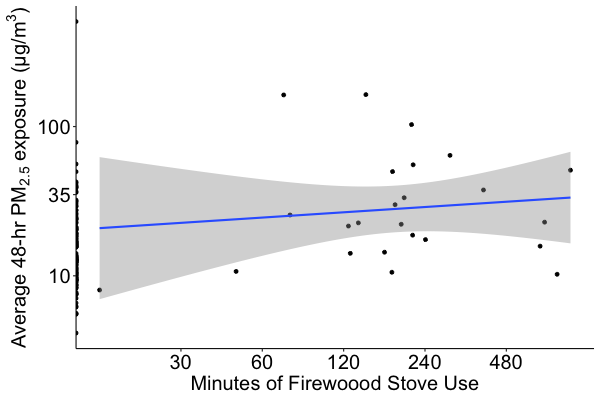 |
| --- |
| **Figure S12. Distribution of the average 48-hr PM2.5 exposure given minutes of firewood stove use.** |

| 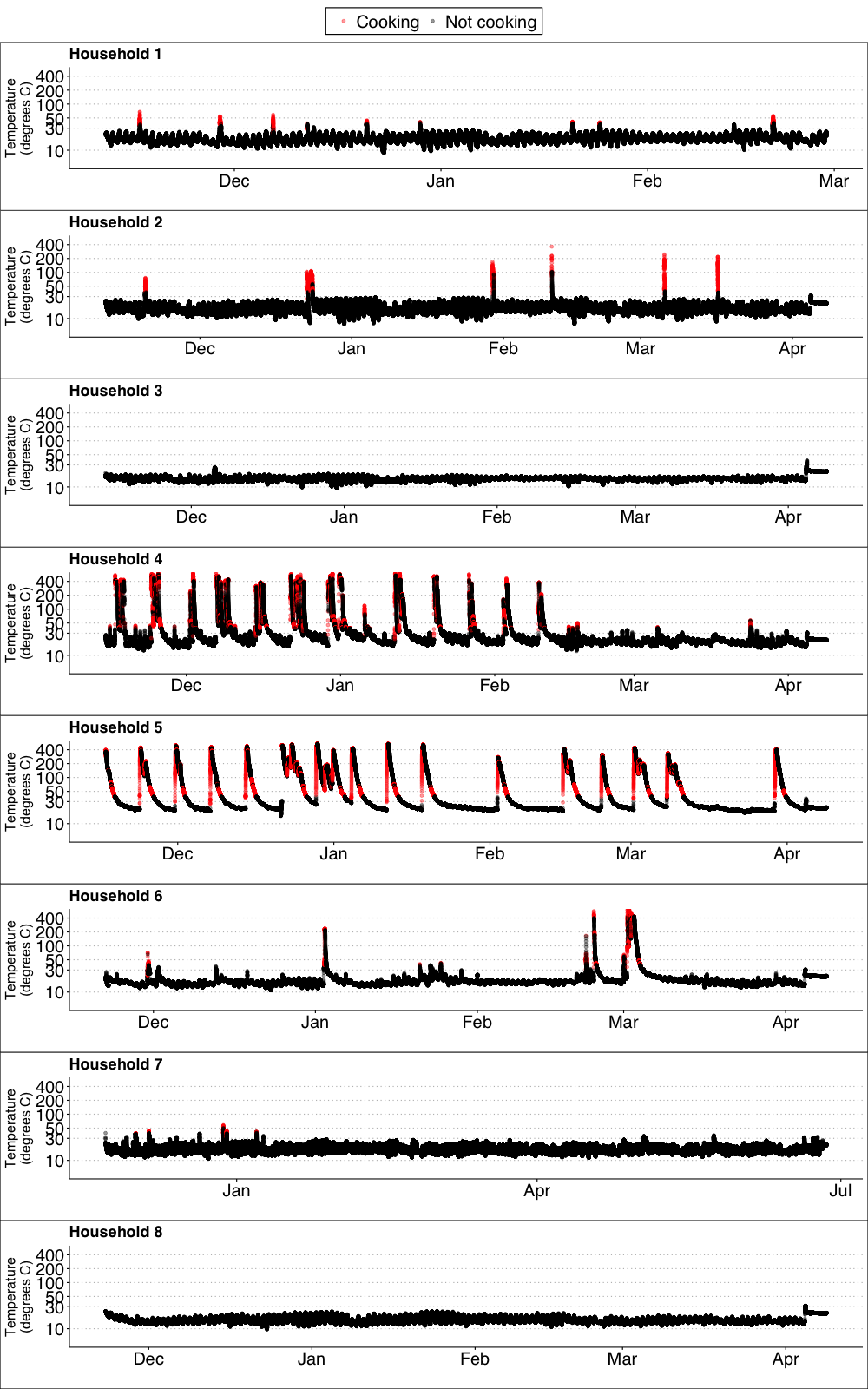 |
| --- |
| **Figure S13. Long-term firewood stove use as captured using Geocene Dots**. Long-term SUMs deployments monitoring the same firewood stove over multiple months. Red points indicate “detected stove use events.” |

| 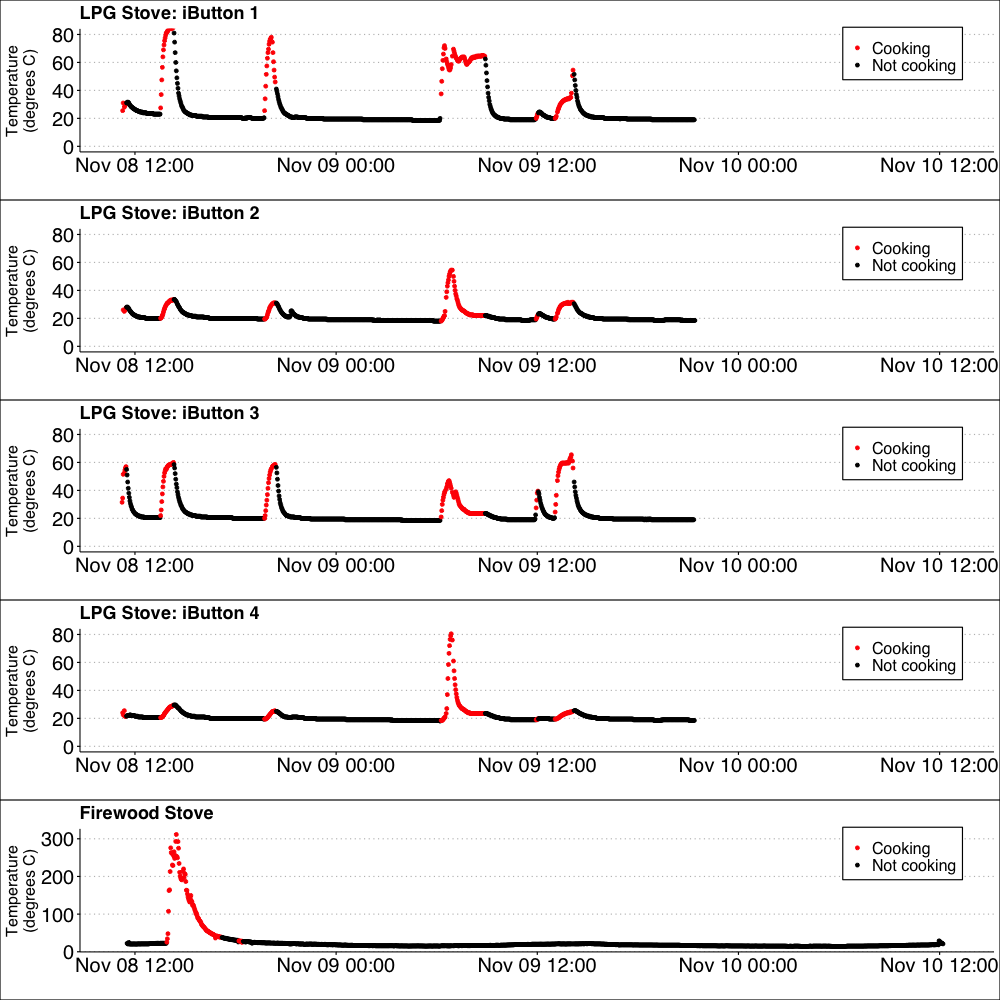 |
| --- |
| **Figure S14. Stove stacking and multiple stove use patterns in a household with a firewood and LPG stove.** Time series data shown are from stove use monitors on a firewood stove and an LPG stove in the same household during the monitoring period. Red dots indicate “detected cooking events.” LPG cooking events are estimated at the “stove level” and therefore shared across iButtons after analysis, regardless of cooking temperature on an individual iButton. |

| 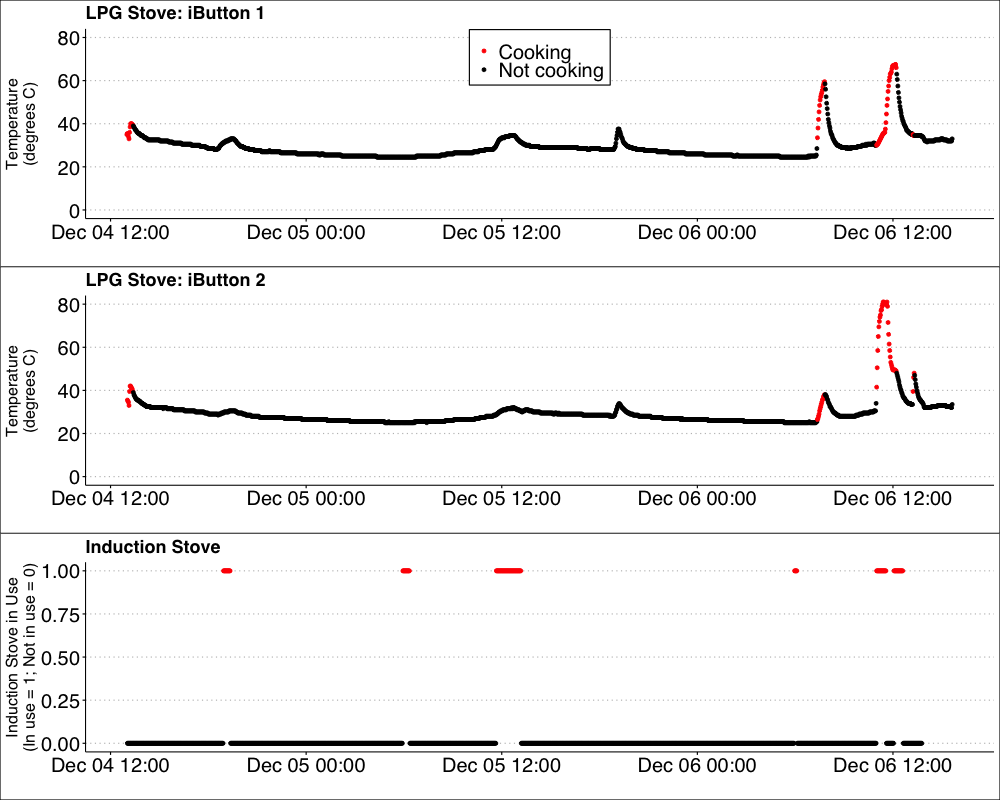 |
| --- |
| **Figure S15. Stove stacking and multiple stove use in a household with an LPG stove and an induction stove. a** For the LPG stove, time series data connect individual temperature data points red dots indicate “detected cooking events.” **b** For the induction stove, a binary “in use” or “not in use” is shown for clarity due to the complexity of interpreting data from the stove use monitor. |

| 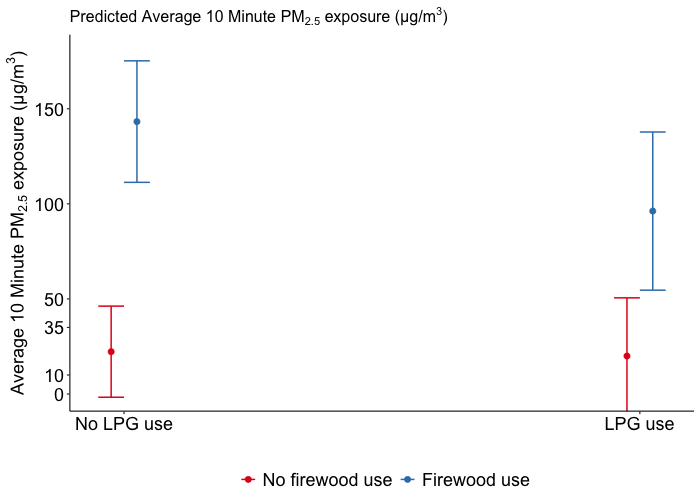 |
| --- |
| **Figure S16. Interaction plot showing predicted average 10-minute PM2.5 exposure when the LPG and firewood stoves are in use.** Estimates derived from Equation 2. Plot generated with the sjPlot package in R. |

| 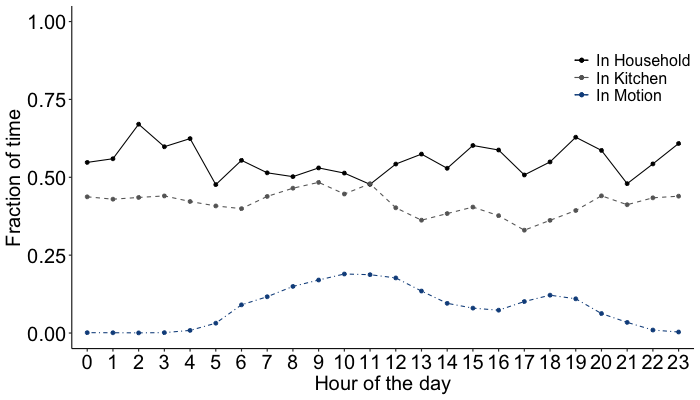 |
| --- |
| **Figure S17. Distribution of the time detected in the household, in the kitchen, or in motion by hour in the day.** |

| 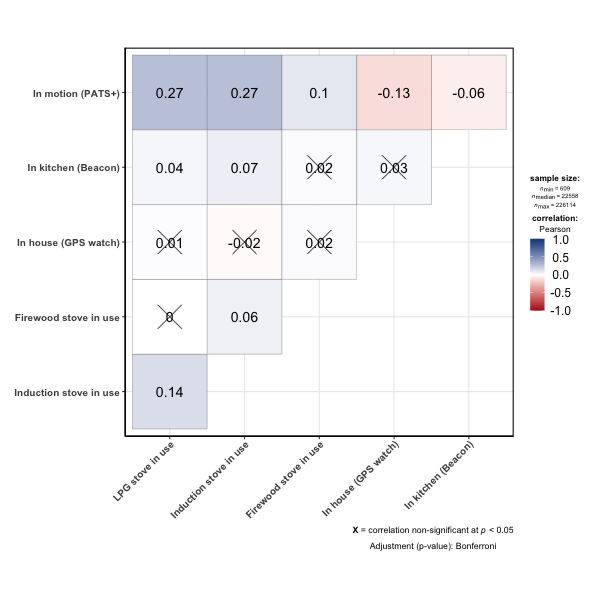 |
| --- |
| **Figure S18. Correlation between detected stove use and participant location.** Correlation plot of detected stove use and participant location in kitchen or in household (method = Pearson; P-Value adjustment for multiple comparisons = Bonferroni). X marks indicate non-significant correlation at P < 0.05, with a Bonferroni correction. |

|  |
| --- |
| **Figure S19. Distribution of daytime compliance during the monitoring period by fuel use categories. a** By primary cooking fuel, **b** By owning a firewood stove, and **c** By owning an induction stove. P-values refer to two-sample t-tests comparing group means. |

| 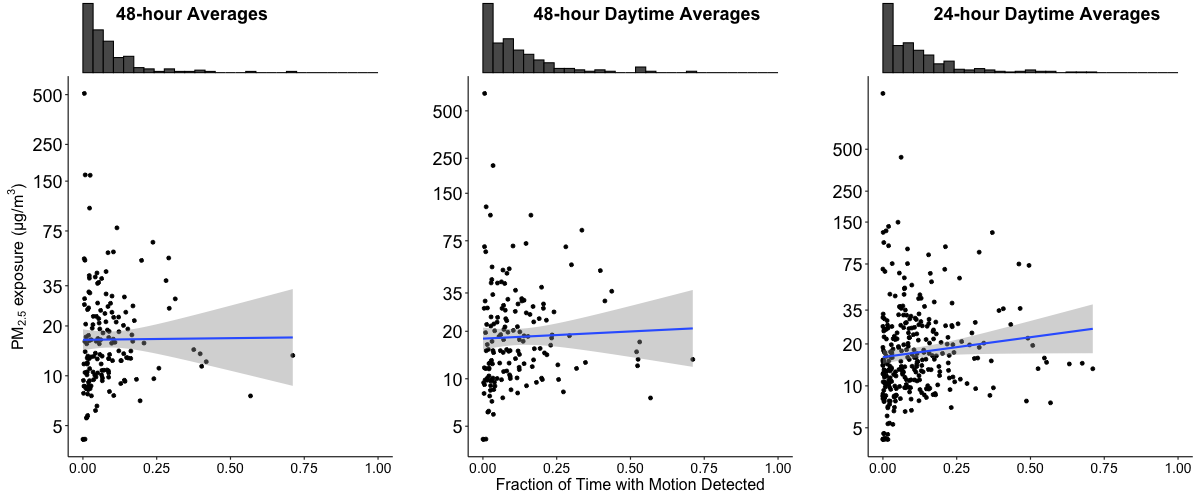 |
| --- |
| **Figure S20. The associations between measures of motion (compliance) and personal air pollution exposure among subsets.** |

| 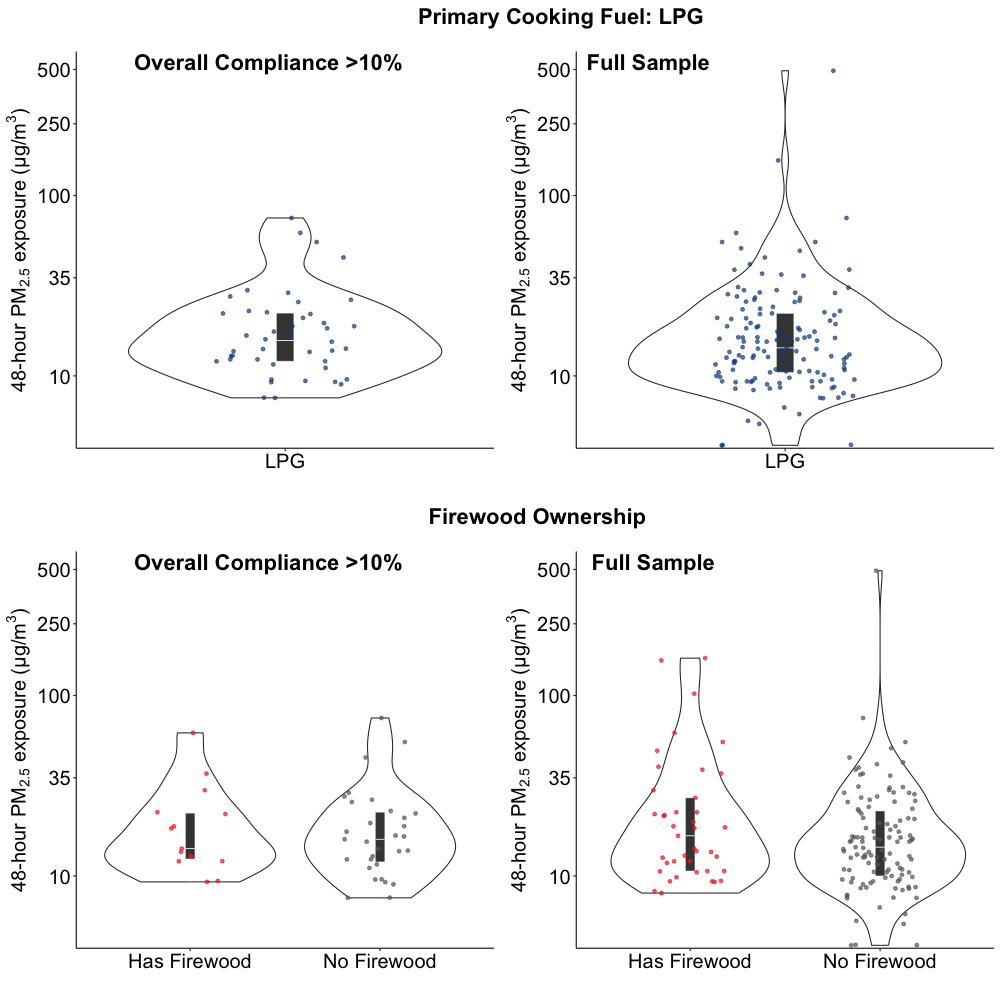 |
| --- |
| **Figure S21. Comparing average 48-hour personal PM_2.5_ exposure in a higher compliance subset of data to the full sample.** Overall compliance refers to the percentage of all observations during the 48-hour period during which there is some recorded motion. The threshold is at approximately 6 hours, which would in our estimation capture most of the cooking across 48-hours. |

| 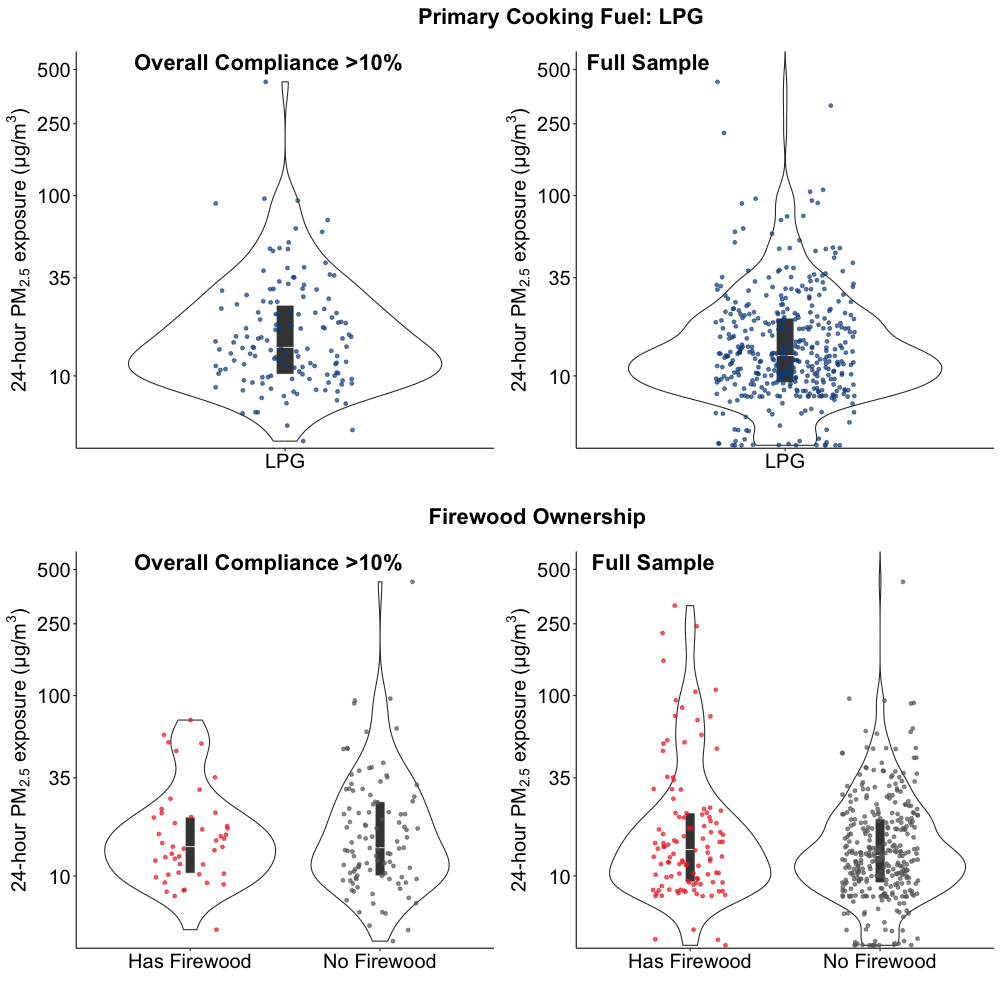 |
| --- |
| **Figure S22. Comparing average 24-hour personal PM_2.5_ exposure in a higher compliance subset of data to the full sample.** Overall compliance refers to the percentage of all observations during the 24-hour period during which there is some recorded motion. The threshold is at approximately 2.4 hours, which would in our estimation capture most of the cooking across 24-hours. |
